# Service models in eating disorders: a scoping review

**DOI:** 10.1186/s40337-025-01252-8

**Published:** 2025-05-19

**Authors:** Rachel Knight, Karen Stagnitti, Genevieve Pepin

**Affiliations:** Deakin University, Geelong, Australia

**Keywords:** Eating disorders, Service models, Anorexia nervosa, Bulimia nervosa

## Abstract

**Background:**

The complexity of eating disorders can be reflected in the different diagnoses, varied clinical presentations and diverse personal circumstances of people living with an eating disorder. Given this complexity, adaptable and flexible service models are essential for effective care. Service models outline a structure for health care delivery that targets the health-related needs of people. The characteristics of existing service models providing assessment and treatment for eating disorders are not well described or understood.

**Objective:**

The purpose of this scoping review was to map and describe the different types of service models for eating disorders and their characteristics.

**Method:**

The JBI methodology for scoping reviews was used. A search of four databases (MEDLINE Complete, EMBASE, PsychINFO, and CINAHL) and grey literature was conducted. Sources describing service models supporting individuals with eating disorders were included.

**Results:**

After duplicates were removed, the remaining sources were screened and read in full, and 30 sources were included in the review. Most service models were eating disorder specialist, located in metropolitan areas and based in community settings. Key characteristics of eating disorder service models included person-centred care, involvement of family and carers, co-design and lived experience contribution, multidisciplinary team, accessibility, identification and management of co-occurring conditions and integration with broader service systems.

**Conclusion:**

Findings indicate vast differences between service models for eating disorders. However, there are examples of innovative and effective service models that show promise. The characteristics of service models for eating disorders identified in this review provided insight into what constitutes an effective and high-quality service model in the sector.

## Abstract (plain language summary)

The purpose of this scoping review was to understand the different types of service models for eating disorders. Service models provide assessment and treatment for people with an eating disorder. In this article, the literature on service models for eating disorders was reviewed to explore what types of service models currently exist and identify their characteristics. There were 30 sources of literature, and all of these were published in academic journals. After reviewing the literature, it was found that service models for eating disorders are very different from each other. However, there were some similarities. For example, most service models for eating disorders are specialist (meaning they only assess and treat people with an eating disorder) and are mostly based in metropolitan areas. The review identified that service models for eating disorders have some key characteristics, and these are; person-centred care, involvement of family and carers, co-design and lived experience contribution, multidisciplinary team, accessibility, identification and management of co-occurring conditions and integration with the service system. Despite the vast differences between service models, innovative and effective examples exist. A clearer understanding of the characteristics of service models is useful for the development of future service models for eating disorders.

## Introduction

The provision of health care is typically offered through service models. Service models are multidimensional and outline a structure for how health care should be delivered [1, 2]. A service model aims to ensure people get the health care they need at the right time and in the right place [1]. The benefit of a clearly articulated service model is consistent and effective health care that aims to sustain or improve health outcomes for a defined group of people [3]. Service models sit within contexts or environments, referred to as service settings. Service models exist across several service settings such as hospitals or the community [4].

Service models and service settings sit within a broader service system. Service systems encompass several interconnected service models across different settings. Ideally, service systems should be coordinated to optimise health outcomes through the delivery of consistent, high-quality health care that promotes the health and wellbeing of the population it serves [3]. An example of a service system being increasingly referenced in eating disorders is a stepped care service system [4, 5]. This service system consists of a range of service models and settings across a continuum of care that offers treatment and support to people with eating disorders. The central premise of the stepped care service system is that a person’s level of need is matched to the most appropriate service model within the system [5].

Eating disorder service systems are complex and vary across countries due to differences in funding, healthcare structures, and population needs. These variations often lead to inconsistent access to care, delayed assessments, and difficulties in starting treatment. As a result, individuals may experience setbacks in their recovery and overall health [6–8]. Given the complexity and severity of eating disorders, it is critical that people can easily access service models that provide quality, evidence-based care because delays can compromise a person’s recovery [9].

A recent rapid review identified eating disorder service models across a range of settings. Findings from this review highlighted the importance of matching the right service model and service setting to a person’s needs [4]. The review recognised the value of service models located in primary care settings to facilitate early detection of an eating disorder. In addition, the review highlighted the important role of community based service models in supporting recovery and improving quality of life. It also found that service models in the community (including day programs) can provide intensive treatment and support, helping to reduce both the number and duration of hospital admissions. While the effectiveness and versatility of eating disorder service models in the community was demonstrated, there was little information about the characteristics of these service models and how they were integrated within the wider service system [4].

Different types of eating disorder service models have been compared with each other in some studies. The Treatment Outcome for Child and Adolescent Anorexia Nervosa (TOuCAN) randomised control trial in the United Kingdom (UK) sought to understand the clinical effectiveness of eating disorder treatment received by young people in hospital inpatient units, community based specialist eating disorder service models, and community based generalist mental health service models (service models that provide assessment and treatment for a variety of mental health conditions) [10]. The results of this study showed no differences between the physical and psychological outcomes of participants (n = 215) across the three service settings. However, a limitation was poor adherence to treatment, particularly in the hospital inpatient units [10]. Similarly, a few years later, health outcomes of young people (n = 378) with eating disorders attending community based specialist eating disorder and generalist mental health service models across 37 services in London were compared [11]. Data revealed that young people in specialist eating disorder service models were more likely to receive a correct eating disorder diagnosis, remain engaged in treatment, and were less likely to be admitted to hospital [11].

Contemporary eating disorder research and guidelines make increasing reference to a stepped care service system [4, 5]. This service system draws together different types of service models, across different settings to create a staged system. Each stage of the stepped care service system has a different capacity to respond and provide a range of evidence-based treatment options and varying levels of support that correspond to a person’s needs. Typically, service models range from least intensive (for example, primary care) to most intensive such as inpatient hospital care [5]. The available literature about stepped care service systems suggests they are more person-centred [12], cost-effective and may optimise clinical outcomes [4], however, more research is required to determine their effectiveness.

Understanding the characteristics of eating disorder service models has been the focus of some studies. One of these studies conducted by Halmi [13] identified, described and recommended the important characteristics of eating disorder service models, across different settings such as community and hospitals. The characteristics that the author recommended, such as the inclusion of a diagnostic and evaluation clinic and a multidisciplinary team, were informed by the available literature, with no primary research conducted. Halmi [13] concluded that despite some characteristics of eating disorder service models having been identified, there was insufficient research currently available to support these recommendations. Another study conducted by Escobar Koch et al. [14] explored essential characteristics of eating disorder service models from the perspectives of 294 participants from the UK and the United States of America (USA). Participants of this study were people who had accessed eating disorder service models in either country. This study found a high-quality, knowledgeable, experienced workforce in eating disorders was paramount for service models. Furthermore, participants expressed that eating disorder service models needed to be easily accessible and offer person-centred and holistic treatment options [14].

Current evidence lacks detail on the characteristics of eating disorder service models, particularly in terms of accessibility and their role in supporting recovery [4, 14]. Several existing documents, including the Australian National Eating Disorder Strategy 2023–2033 [5], Canada’s Clinical Practice Guidelines for the BC Eating Disorders Continuum of Services [15] and Ireland’s Eating Disorder Services HSE Model of Care [16] outline stepped care service system frameworks for eating disorders, detailing their structure, components and functions. These frameworks for stepped care service systems provide a high-level, broad structure on how assessment and treatment for an eating disorder can be accessed. However, these documents do not describe service models within the service system or their characteristics. Therefore, a more detailed and specific articulation of service models is warranted to provide more consistency between service models and identify what these service models need to optimise the provision of support and care to people with eating disorders.

By definition, scoping reviews explore the breadth of research on a topic [17]. Their purpose includes clarifying key concepts in the literature, identifying key characteristics related to a concept and identifying and analysing knowledge gaps [17–19]. Therefore, a scoping review seemed the most appropriate way to detail the different types of service models in eating disorders, highlight the key characteristics of service models and examine the variations between these. Scoping reviews are the most useful approach to map the depth and breadth of a topic or concept, especially those that are emerging or for which there is limited existing evidence [20]. In addition, scoping reviews are most appropriate for mapping the available literature [21, 22] and do so by including diverse forms of evidence [20]. Therefore, this scoping review was undertaken to explore the variety of eating disorder service models described in the literature, drawing from multiple sources to address the following questions:

1. What are the different types of service models for eating disorders?
2. What are the characteristics of eating disorder service models?

## Methods

The JBI methodology for scoping reviews [21, 22] was used to describe and map the different types of service models for eating disorders and their characteristics. Further adding to the rigour of the methodology, the quality indicators from the Preferred Reporting Items for Systematic Reviews and Meta-Analyses extension for scoping reviews guidance (PRISMA-ScR) [23] were used.

### Inclusion and exclusion criteria

In this review, any literature source and study design were considered for inclusion. The ‘PCC’ mnemonic guides scoping reviews and stands for ‘Person’, ‘Context’ and ‘Concept’. In this scoping review, literature sources were included if they focused on an eating disorder population (person) and described a service model (context) and its characteristics (concept). Sources were included if they were written in English and published between 2010 and 2024. These search dates were decided as 2010 was when most literature on service models for eating disorders began to be published, capturing 15 years of literature on the topic.

Any source that did not meet the inclusion criteria, had an intervention focus or did not describe a service model (for example, described a service system, such as a stepped care service system) or the characteristics of a service model were excluded.

### Search strategy

A comprehensive search strategy was conducted in May 2024 and updated in September 2024 with electronic databases MEDLINE Complete (EBSCOhost), EMBASE, PsychINFO, and CINAHL. Relevant subject headings for each of the databases were also included. A list of search terms is presented in Table 1. A search of grey literature was conducted broadly on the internet using the search terms to identify guidelines, government reports, and protocols. The authors also searched the websites of several national and international eating disorder organisations and relevant government bodies.

**Table 1:** Scoping review search terms

Concept 1—Eating Disorders	Concept 2—Service Model
“Eating disorder*” “Disordered eating” Anorexi* “Anorexia nervosa” Bulimi* “Bulimia nervosa” EDNOS “Binge eating disorder” “Avoidant restrictive food intake disorder” ARFID “Other specified feeding or eating disorder” OSFED “Unspecified feeding or eating disorder” “Atypical anorexia nervosa”	“Service model” “Model of care” “Continuum of care” “Service system” “Service delivery” “System of care” Service* “Care pathway”

**Fig. 1:**
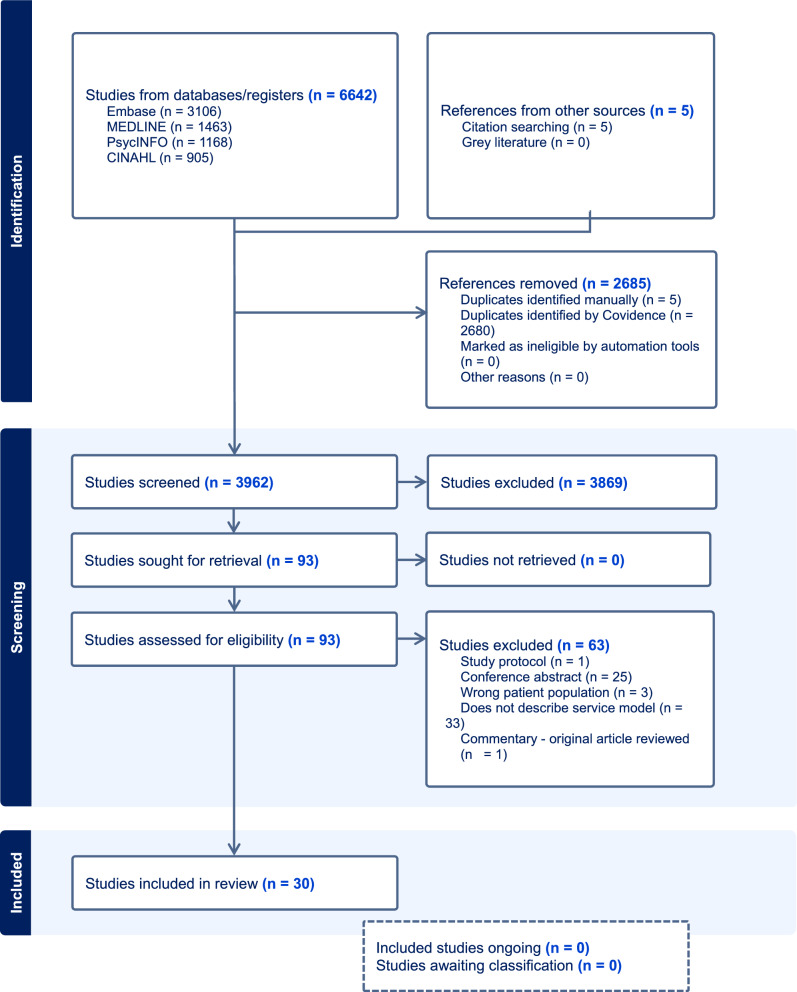
PRISMA flow chart

A total of 6642 individual sources were initially identified. After duplicates were removed, 3957 remained. Titles and abstracts were screened by RK against the inclusion criteria using Covidence Systematic Review Software and 3869 sources were rejected because they did not meet the inclusion criteria. If there was uncertainty about whether a source met the inclusion criteria, GP was consulted. A total of 88 sources were read in full. All full text sources were independently reviewed by two authors (RK and GP). Of these 25 were included in the scoping review. Discrepancies were discussed before reaching a consensus. Reviewing reference lists revealed a further five studies that were included because of their relevance to the review questions. No grey literature sources were located that met the inclusion criteria. Therefore, a total of 30 literature sources were included. Detailed information can be viewed in Fig. 1.

### Data extraction

In line with recommended best practice [24], a table was developed to ensure key criteria about the service models were extracted from the data in a standardised way. Data describing the different types of service models were extracted and included the country of data collection, if the service model was eating disorder specialist (or not), the service model setting and location, and the population targeted by the service model. If the literature source included research or evaluation of the service model, further data were extracted such as participant demographics, aims, study design, and outcomes. This information is detailed in Table 2. Data was also extracted on the characteristics of the service model. Determining the criteria for this part of the extraction tool was informed by the National Eating Disorders Collaboration (NEDC) National Standards for Eating Disorders practice principles [25], which form the foundation for an effective and consistent approach to eating disorder care. Characteristics of service models encompassed; person-centred care, involvement of family and carers, co-design and lived experience contribution, the multidisciplinary team, accessibility, identification and management of co-occurring conditions and integration with the service system. The characteristics are described in Table 3. During the data extraction process, iterative modifications were made to the table to ensure it captured the most relevant criteria that aligned with the review questions [19].

**Table 2:** Types of eating disorder service models

Author/Year/ Country	ED specialist	Service setting	Service location	Population targeted	Research aims	Research design	Demographics of participants	Research findings/ outcomes
Allen, Mountford, Brown, Richards, Grant, Austin, Glennon & Schmidt (2020) UK	Yes	Community/ early intervention	All	Young adults (aged 16-25 years old) with a primary diagnosis of an eating disorder <3 years in duration	-	-	-	-
Anderson, Desai, Zalaznik, Zielinski & Loeb (2021) USA	Yes	Community	Metropolitan	People with an eating disorder across the lifespan	-	-	-	-
Bern, Milliren, Tsang, Mancini, Carmody, Gearhart, Eldredge, Samsel, Crowley & Richmond (2024) USA	Yes	Hospital	Metropolitan	People with a diagnosis of ARFID aged between 4 and 21 years old	The study had three aims: To examine the variability in care for people with ARFID across three inpatient units in the same hospital (Adolescent/Young adult Medicine, Gastroenterology and General paediatrics). To create a standardised inpatient clinical pathway. To examine changes in care for inpatients with ARFID post the inpatient clinical pathway compared to before initiation of inpatient clinical pathway.	Quantitative: Retrospective review	Two groups of participants; n=57 people pre- inpatient clinical pathway and n=53 people post-inpatient clinical pathway. All participants were between 4-and 21 years old, with a diagnosis of ARFID. Overall, participants were mostly female (61.8%) and race classified as white (76.4%). Ethnicity was non-Hispanic (78.2%), Hispanic (7.3%) and unknown (14.5%). SES not reported but 70.9% of participants had private health insurance.	Development of a three step inpatient clinical pathway was completed and implemented. Following implementation of the inpatient clinical pathway variability in case reduced. More specifically, there was improved consultation between social work, nutrition and psychiatry. There was a minimal reduction in length of stay from 7 days to 6.2 days (on average).
Brown, McClelland, Boysen, Mountford, Glennon & Schmidt (2016) UK	Yes	Community/ early intervention	Metropolitan	Young adults (aged 18-25 years old) with a primary diagnosis of an eating disorder <3 years in duration	To assess the feasibility and acceptability of the First Episode and Rapid Early Intervention for Eating Disorder Service (FREED) on reducing the duration of time until specialist service contact, duration of untreated eating disorder and waiting time compared with previous practice within the service	Quantitative: repeated measure design	Two groups of participants: 1) FREED cohort = 51 people with a primary diagnosis of an eating disorder for three years or less. Participants were aged between 19-25 years old 2) Audit cohort = 89 people with similar diagnosis, age and illness duration	There was a slight reduction in duration of time between specialist service contact and duration of untreated illness when compared to previous practice. Reduced waiting times were achieved through the implementation of the FREED service
Bryant-Waugh, Loomes, Munuve & Rhind (2021) UK	Yes	Community	Metropolitan	Children and Adolescents with a diagnosis of ARFID	To describe and share an evidence informed, multidisciplinary, multi-model outpatient care pathway for young people experiencing ARFID (aged between 2-17 years).	-	3 case examples of young people aged between 4-17 years old with a suspected diagnosis of ARFID 67% female, 33% male. Ethnicity reported as one white British, one Pakistani British, and one black Caribbean. Race and SES not reported.	The framework could offer a structure to support clinicians in guiding assessment and treatment of young people with an ARFID diagnosis, until further research is conducted in the area.
Clinton, Almlof, Lindstrom, Manneberg & Vestin (2014) Sweden	Yes	Community	Regional	Adults with an eating disorder	To explore the possible advantages and disadvantages of drop in access to the treatment of eating disorders	Qualitative: semi-structured interviews	11 people with an eating disorder diagnosis. Participants aged between 19-34 years old. 100% female, from a working- or middle-class background	Participants reported that the drop-in program helped overcome their fear of treatment, increased accessibility and allowed participants to feel secure and safe in the program environment
Dror, Kohn, Avichezer, Sapir, Levy, Canetti, Kianski & Zisk-Rony (2015) Israel	No	Hospital	Metropolitan	Children and adolescents with psychiatric conditions (including eating disorders)	To evaluate treatment success of four-phased reintegration guidelines following hospitalisation	Mixed Methods: cross sectional design (using interviews and a review of medical charts)	Six adolescents and their parents, plus an additional seven parents only (total 13 participants). There were 11 female and 2 male patients. Medical records data for 11 of the 13 participants were used in the evaluation. Seven patients were hospitalised for AN and six for EDNOS. Age ranges between 12.5 – 17 years. 11 parents were married and two were divorced. Two other families were identified as complex.	Eleven young people successfully reintegrated back into the educational system. BMI increased from admission to discharge. Twelve were receiving ongoing treatment for their eating disorder in the community.
Eisler, Simic, Fonagy & Bryant-Waugh (2022) UK	Yes	Community	All	Children and adolescents with an eating disorder (of any severity)	-	-	-	Research was not presented; however, the authors included some brief outcomes from the ongoing evaluation citing reduced wait times and increased identification and referral of eating disorders
Fenner & Kleve (2014) UK	Partially	Community	Regional	Children and adolescents	To describe the service model and outcome evaluation of an eating disorder service model based on a community Child and Adolescent Mental Health service	Quantitative: retrospective audit of case files (demographic data, family composition, family involvement in treatment, medication administered, treatment length, inpatient admissions, if menstruation was present, if vomiting was present, height, weight and BMI). Service user feedback was obtained via a questionnaire	45 children and adolescents with a diagnosis of AN or EDNOS. Females made up 93% of participants. Most participants were from an intact two-parent family (n=36) Ethnicity was recorded for 25 cases, all were White British Race and SES was not reported	Treatment within the service mostly involved families (n=41, 98%), and 31 people received both individual and family work (76%). Three cases dropped out of treatment and a further one was referred to adult services. Authors reported significant improvements against Morgan Russell outcomes for most participants (AN = 77% and EDNOS = 82%) Service satisfaction ratings were high
Goldstein, Peters, Baillie, McVeagh, Minshall & Fitzjames (2011) Australia	Yes	Community -intensive	Metropolitan	Adolescents aged between 12-18 with an eating disorder	To determine the effectiveness of a day patient program for the treatment of adolescents with AN or EDNOS	Quantitative: multiple measure research design	26 adolescent females with a diagnosis of AN or EDNOS Race, SES, and ethnicity not reported.	Significant changes were achieved following treatment on measures of weight gain. Some positive changes in behaviour and attitude.
Hayes, Tweedy & Chapman (2024) UK	Yes	Community - intensive	Metropolitan	Young people aged between 10-18 with a diagnosis of AN	The study aimed to evaluate the effectiveness of a modification in service model and treatment approach from a group based programme to an intensive family based program.	Quantitative: retrospective chart review	Young people (n=190) with AN who accessed the service between March 2017 and April 2023. These were split into two groups, depending on which version of the intensive program they accessed: Original Model n=86 (female n=85, male n=1) New Model n=104 (female n=97, male n= 7) Race, ethnicity and SES were not reported.	The new service model resulted in a significant reduction in length of admission from 135 days (original model) to 89 days (new model). There was also a reduction in inpatient admissions after implementation of the new service model, from 29 people (original model) to 11 people (new model).
Herpertz-Dahlmann, Borzikowsky, Altdorf, Heider, Dempfle & Dahmen (2021) Germany	Yes	Community - intensive	Regional	Children and Adolescents with AN or AAN, who have received treatment on an inpatient unit	To pilot an eating disorder specialist home treatment and to investigate its feasibility, effects, and safety	Quantitative: single centre nonrandomised open-label pilot study	22 young people with a diagnosis of AN or AAN, who had received inpatient treatment for their eating disorder. All participants were female, 20 (90.9%) lived with both parents, and 2 (9.1%) lived with one parent/patchwork family. 18 (81.8%) had a psychiatric co-occurring condition.	Eating disorder symptoms, general psychopathology and weight improved considerably whilst receiving treatment in the service model. Carers skills also increased, and carer burden decreased. Both young people and carers reported high levels of satisfaction with the service model. There was no safety concerns associated with the service model.
Johnson, Cook, Cadman, Anderson, Williamson & Wade (2022) Australia	Yes	Community	Regional	People with an eating disorder aged 14 years old or above	To evaluate and report on the outcomes of a non-specialist community-based service model for a regional area	Quantitative: case series	143 people who met the DSM-5 diagnostic criteria for an eating disorder (diagnoses not reported, but 21% reported to have a BMI <18.5)	Dropout rates were low, with 103 participants completing treatment (15 transferred to alternative pathways and 25 cases dropped out). Participants experienced significant improvements in eating disorder cognitions. Underweight participants, 36.4% of completers achieved a normative BMI.
Kaplan, Hutchinson, Hooper, Gwee, Khaw, Valent & Willcox (2024) Australia	No	Hospital	Metropolitan	People with a primary mental health diagnosis and a co-occurring eating disorder (AN BN, OSFED) or significant disordered eating behaviours	The aim of the study was to evaluate the implementation of a multidisciplinary, evidence informed screening and care pathway for people with a co-occurring eating disorder on general inpatient units	Mixed Methods: file audit and interviews, mapped to the RE-AIM framework	Audits of 632 patient files (pre and post pathway implementation) were conducted. 65% (n=411) were female and 3.3% (n=21) had an eating disorder diagnosis. Age, race, ethnicity and SES were not reported. In addition, 7 people with a co-occurring eating disorder (All female, diagnosed with AN [n=5], OSFED [n=1] and disordered eating behaviours [n=1]) and 18 clinicians were interviewed. Clinicians were from a range of disciplines, including nursing, allied health and psychiatry. No further demographics such as age, gender, race, ethnicity or SES were reported. 50 clinicians (27 mental health nurses, 19 allied health and 4 intake clinicians) completed the training modules. No further demographics were reported.	Identification of disordered eating behaviours was three times more likely (OR =3.3, 95% CI (1.6-7.1) *p*=.002) following implementation of the new service model. People with an eating disorder were also significantly more likely to be referred to a dietitian and more structured person-centred care. An eating disorders pathway can feasibly and successfully be implemented into an existing general mental health inpatient unit.
Milton, Hambledon, Dowling, Roberts, Davenport & Hickie (2021) Australia	Yes	Telehealth	All	Across the lifespan	To customize and configure a technological, non-traditional service model (web-based, phone, email) that provides support for eating disorders and body image issues	Qualitative: participatory design	45 people aged 15 or older across six workshops. Participants had a lived experience of eating disorders, disordered eating, body image and related issues (n=13), significant other (n=7), health professionals with a lived experience of an eating disorder (4), health professionals (n= 21) Gender, race, SES, and ethnicity not reported	Participants highlighted that there is a critical need to address some of the barriers to care. Seven themes were identified from the workshops: 1. identified barriers to care within the current system; 2. need for people to be able to access the right care anywhere, anytime; 3. recommendations for the technological solution (i.e., InnoWell Platform features and functionality); 4. need for communication, coordination, and integration of a technological solution embedded in Butterfly's National Helpline; 5. need to consider engagement and tone within the technological solution; 6. identified challenges and areas to consider when implementing a technological solution in the Helpline; 7.and potential outcomes of the technological solution embedded in the Helpline relating to system and service reform.
Moron-Nozaleda, Yanez, Camarneiro, Gutierrez-Priego, Munoz-Domenjo, Garcia-Lopez, Garcia, Garcia, Trujillo, Faya & Graell (2023) Spain	Yes	Community – intensive	Metropolitan	Children and Adolescents with an eating disorder (AN, ARFID, BN, AAN, OSFED), who required hospitalisation as a result of their eating disorder.	To explore the feasibility and acceptability of a Hospital in the Home program for adolescents with a severe eating disorder	Quantitative: Retrospective data collection (demographics, clinical variables, chart reviews) and a satisfaction survey (for families of participants) compromising 13 questions with a Likert scale for response	59 females (mean age = 14.69 years). Diagnosis was Restrictive AN (n=30), AAN (n=7), Purging AN (n=3), BN (n=5), ARFID (n=3) and OSFED (n=11) 28 participants had one or more cooccurring condition and 47 participants lived with both parents, while the parents were divorced for 12 participants. Race, SES and ethnicity not reported.	Authors concluded that the service model was feasible and had a high retention rate (90%). Families of participants (n=43) were very satisfied with the service model (score 4.95/5, SD = 0.5) and perceived the model as ‘very safe’.
Munro, Thomson, Corr, Randell, Davies, Gittoes, Honeyman & Freeman (2014) UK	Yes	Community - intensive	Regional	People with an AN. Age range targeted not reported.	To describe a service model for eating disorders and present primary evidence of the program efficacy	Mixed Methods: cross sectional survey design	Not described	Overall, participants were satisfied with the service. Qualitative data showed that staff were perceived to be supportive, caring and genuine. In addition, participants valued the holistic approach to treatment and individualised care.
Newell (2023) UK	Yes	Community	Regional	Across the lifespan	-	-	-	-
Newton, Bosanac, Mancusa & Castle (2013) Australia	Yes	Community and community intensive	Metropolitan	Adults (aged 16 years +)	To report on the development of a community eating disorder specialist program and its initial 18-month evaluation	Quantitative: pre-post design	208 people with an eating disorder who had attended the program. Participants aged 16+ years old. There were 188 (90.4%) females and 20 (9.6%) males. Only 197 of the 208 participants were included in the analysis (as there were 11 unplanned discharges), however the genders of this smaller group were not reported. Most had a diagnosis of AN (n=97, 53.3%), BN (n=41, 22.5%) and EDNOS (n=43, n=23.6) For the day program, there were 47 participants, 43 females (91.5%) and 4 males (8.5%) and 80.6% were aged under 25 years old. Diagnosis included AN (n=36, 76.6%) and BN (n=6, 12.8%) and EDNOS (n=5, 10.6%). Race, SES and ethnicity not reported	Attendance at the community service model led to statistically significant differences between baseline and follow up scores on a range of self-report measures that showed improvements in eating disorder symptoms, quality of life, and mood symptoms. Increased BMI and weight (for people this was indicated for) also achieved.
Painter, Ward, Gibbon & Emmerson (2010) Australia	Yes	Community, community intensive and hospital	Metropolitan based, servicing all locations (metropolitan, regional and rural)	Adults with an eating disorder	-	-	-	-
Penfold (2015) UK	Yes	Community - intensive	Regional	Adults with a diagnosis of AN (aged 16+)	-	-	-	Anecdotal reports of lower admission rates, improved eating disorder behaviours and increased weight
Simic, Stewart, Eisler, Baudinet, Hunt, O’Brien & McDermott (2018) UK	Yes	Community - intensive	Metropolitan	Adolescent aged between 11-18 years old with a restrictive eating disorder diagnosis	There were two study aims: To report of participant outcomes following a short day program And to describe the longer-term outcomes of the participants, 6 months following completion of the program and discharge from the outpatient eating disorder service model (within the wider service system)	Quantitative: retrospective uncontrolled case series	Adolescents aged between 11-18 years old (n=105). Predominantly females (95.2%) and white British ethnicity (88.6%). Race and SES not reported.	Following attendance at the day patient program, young people reported lower levels of depression and an increased ability to regulate emotions. In addition, there was improved self-esteem, quality of life and motivation. Participants achieved weight gain and a reduction in eating disorder pathology. Most participants in the day patient program went on to successfully complete outpatient treatment and 73% achieved a good or intermediate outcome
Strand, Gustafsson, Bulik & Hausswolff-Juhlin (2015) Sweden	Yes	Hospital	Metropolitan	Adults with longstanding eating disorders	-	-	-	-
Suetani, Yui & Batterham (2015) Australia	Yes	Hospital	Metropolitan	Children with an eating disorder	-	-	-	-
Tantillo, Starr & Kreipe (2020) USA	Yes	Telehealth	All	Across the lifespan	To describe an innovative tele mentoring project and evaluate the programs acceptability, practitioner satisfaction, and impact on knowledge gained and intended practice changes.	Mixed methods: questionnaire and survey	99 health professionals completed the Project ECHO Eating Disorders Clinic outcome questionnaire, and 30 health professionals completed the Continuing Medical Education surveys Gender (of those who reported) Female n=54 (94.6%) Ethnicity (of those who reported) White n=57 (87.7%) and of non-Hispanic origin n=61 (95%) Race and SES was not reported	Female social workers were the most frequent attendees of the program. Participants predominantly worked in primary care or outpatient settings. Participants agreed that the project objectives were met, information was balanced, and evidence based, organised, clear and relevant to stated objectives. In addition, nine themes were identified in the content analysis. Of these, four related to key points learned (1. Eating disorders are complex illnesses, 2. Attending to team relationships, 3. Motivating patient for change and 4. Essential elements of care) and five related to changes you would make in your practice (1. Improving screening practices, 2. Motivating patients for change, 3. Improving treatment, 4. Attending to team relationships and 5. Seeing and treating the whole patient)
Tchanturia, Smith, Glennon, & Burhouse (2020) UK	Yes	Hospital	Metropolitan	Adults with a diagnosis of AN and Autism	-	-	-	-
Wallis, Alford, Hanson, Titterton, Madden & Kohn (2013) Australia	Yes	Hospital	Metropolitan	Families with a young person diagnosed with AN, who were from rural areas OR vulnerable families and those with a poor response to outpatient FBT	-	-	-	-
Weber & Davis (2012) Australia	Yes	Community	Rural	People over the age of 14 years old with a suspected eating disorder	To examine the effectiveness of an assessment and referral model of eating disorder service delivery and its potential utility as a model for rural service delivery	Qualitative: evaluative design	Brief semi-structured interviews n=40 clients of service (2004-2006); extended semi-structured interviews n=4 clients of service; surveys, 2 time points (2005) n=14 & (2006) n=21 with service providers; semi-structured interviews n=20 parents; 2007 & 2008); written survey n=12 parents/carers; (2007); brief semi-structured interviews n= 27 clients and parents (2007); extended semi-structured interviews n=28 service providers (2007 & 2009) All client participants (except one) were female. Race, SES and ethnicity not reported	Eating disorder assessment was useful, however there was numerous challenges in finding local treatment options. Challenges included a lack of clinicians with expertise in eating disorders and a lack of experienced eating disorder clinical supervisors
Williams, Dobney & Geller (2010) Canada	Yes	Community	Metropolitan	People with longstanding eating disorders, when recovery focused treatment was shown to have limited impact	To present preliminary outcome research of a community outreach partnership program for people with eating disorders	Quantitative: pre-post design	31 people with an eating disorder who accessed the program for at least four months in duration. Participants has a diagnosis of AN (n=15, 48%), BN (n=3, 10%) and EDNOS (n=13, 42%) Mean age of participants was 31.07 years old and duration of eating disorder on average was 15.23 years. Gender, race, SES and ethnicity not reported	Significant improvements were seen in global distress scores, hopelessness, body mass index and eating disorder symptoms between starting and completing program. Participants reported that they had improved relationships, decreased importance about weight and shape and determinants of self esteem
Williams, O’Reilly & Coelho (2020) Canada	Yes	Community - intensive	Metropolitan	Transition age youth (16-24 years old)	To understand the clinical characteristics of residents at the residential service model, examine predictors of early treatment and explore residents’ perception and experiences with residential treatment.	Mixed Methods: retrospective review of medical records and qualitative analysis of interviews conducted with residents who had received treatment (for the full duration) from the service model	Retrospective chart review n=193 Most were females (n=186, 96.4%), with less males (n=4, 2.1%) and some participants did not identify their gender or identified and gender nonbinary (n=3, 1.6%). Most participants had a diagnosis of BN (n=73, 37.8%) or AN restricting type (n=34, 26.9%). Other diagnoses included AN binge-purge type, AAN, atypical BN, EDNOS and BED. Qualitative interview n= 39 Most participants were female (n=38, 97%) and one person identified as gender neutral (n=1, 3%). Race, SES and ethnicity not reported	Results from the chart review showed that participants (n=85) who were underweight (<BMI 20) at the start of treatment had a significant increase in weight at discharge. Qualitative analysis of the interviews found that 35.9% of interviewees self reported no longer engaging in eating disorders behaviours at discharge. A further 48.7% reported improvement in their behaviours since admission. Eating disorder thoughts were also reported to be less frequent (n=31, 79.5%). Thematic analysis identified what participants perceived to be the most helpful aspects of the service model: a) the benefit of structured eating and gradual exposure to increasing nutritional responsibility and challenges, b) the utility of individual therapy, c) the benefit of therapeutic groups and d) the importance of a multidisciplinary approach

*AN* Anorexia nervosa, *AAN* Atypical anorexia nervosa, *ARFID* Avoidant restrictive food intake disorder, *BED* Binge eating disorder, *BN* Bulimia nervosa, *BMI* Body mass index, *DSM V* Diagnostic statistical manual of mental disorders fifth edition, *EDNOS* Eating disorder not otherwise specified, *FREED* First Episode and Rapid Early Intervention for Eating Disorder Service, *OSFED* Other specified feeding and eating disorder, *SES* Socio economic status

**Table 3:** Eating disorder service model characteristics

Author/Year/ Country	Person-centred care	Involvement of family and carers	Co-design and lived experience contribution	Multidisciplinary Team	Accessibility	Co-occurring conditions	Integration with the service system
Allen, Mountford, Brown, Richards, Grant, Austin, Glennon & Schmidt (2020) UK	A ‘commitment’ to person-centred care.	Is encouraged.	A group of young people who have benefitted from or want to support FREED has been established, who provide input into new initiatives for the service model and promotion of the service model.	FREED site-based champion is an important part of the service model. No further details of the team reported.	Service model is for people aged between 16-25 years old with an eating disorder with a duration of <3 years. All referrals receive an engagement phone call within 48 hours of referral. This phone call validates help seeking, screens for eligibility for the service model and provides initial information about early intervention. Then, a person should receive an assessment within 2 weeks, and commence treatment within 4 weeks of referral.	-	Service model is considered a ‘service within a service’, meaning it sits within a wider eating disorder service system.
Anderson, Desai, Zalaznik, Zielinski & Loeb (2021) USA	-	-	-	Service model team includes 12 therapists (disciplines not reported), and this team collaborates with healthcare providers in psychiatry, paediatrics, adolescent medicine, gastroenterology, speech therapy and occupational therapy. Minimum care team is a primary therapist and an external physician. Team receives group consultations and one-to-one supervision and has access to expert external consultation (for support with complex cases).	Intake process is aligned with the fundamental tenets of treatment, ensuring intake calls are completed within 24 hours (if possible), and people are referred to other services rather than being placed on a waiting list for treatment. Intake calls are also used to provide psychoeducation.	Service model does address co-occurring psychiatric conditions using treatment models such as DBT and exposure and response prevention.	Service model reported to have established networks with hospitals and medical providers in the area.
Bern, Milliren, Tsang, Mancini, Carmody, Gearhart, Eldredge, Samsel, Crowley & Richmond (2024) USA	-	Family and carers were involved in step two of the pathway, particularly with meal support. Parents and carers also had access to psychoeducation from the care team and were involved in discharge planning.	-	Included specialists in adolescent/ young adult medicine, gastroenterology, psychology, psychiatry, nutrition, social work and nursing.	Any person admitted with a diagnosis of ARFID. People were eligible if they were between 4 and 21 years old.	It was reported that over half of the participants had a co-occurring condition such as depression and anxiety. No treatment for co-occurring conditions was reported.	-
Brown, McClelland, Boysen, Mountford, Glennon & Schmidt (2016) UK	Aimed to deliver person-centred care. Care plans were collaboratively developed.	Actively encouraged family and carers to participate in assessment and treatment.	-	-	Inclusion criteria: age range of 18-25 years old, a primary eating disorder diagnosis and an eating disorder illness duration of <3 years. Exclusion criteria: the need for immediate inpatient admission and/or a severe learning disability or co-occurring condition requiring treatment. Referrals were encouraged from primary care and planned to accept self-referral in future. All referrals received a screening telephone call within 48 hours. If they are deemed eligible for service model, they were booked into an assessment (aiming for <2 weeks from referral date).	Cooccurring conditions that required treatment were exclusion criteria.	Service model is considered a ‘service within a service’, meaning it sits within a wider eating disorder service system.
Bryant-Waugh, Loomes, Munuve & Rhind (2021) UK	ARFID formulation, goals and treatment plan developed in collaboration with the young person and their family.	Parental involvement is encouraged at all stages and varies between young people. At a minimum, parents and carers actively support the young person between sessions to engage in therapeutic tasks linked to agreed goals.	-	Multidisciplinary team mentioned - including occupational therapists, speech and language therapists.	Aims to remove any barriers to referral, recognising that referrals may come from a wider range of clinicians and services than other eating disorder diagnoses. Referrals are screened to determine the urgency of assessment and confirm that the presentation doesn’t fit any other eating disorder diagnosis. Self and parent referrals are also accepted.	Acknowledgment of common co-occurring conditions (such as Autism) and noted that this is discussed as part of the assessment. Treatment for co-occurring conditions not reported.	Service model sits within the wider eating disorder service system.
Clinton, Almlof, Lindstrom, Manneberg & Vestin (2014) Sweden	Drop-in program enables people to attend the service with no appointment, no obligations and no expectations.	-	-	12 member multidisciplinary team: psychiatric nurses, clinical psychologists, a social worker, a psychiatrist and a physician.	Drop-in sessions aimed to enable earlier intervention. Two-thirds of people receiving treatment from the service accessed the service via the drop in sessions. Also received referrals from other parts of the health service. No eligibility criteria, open to any adult who feels a need for the service.	-	Positioned within a wider publicly funded eating disorder service system.
Dror, Kohn, Avichezer, Sapir, Levy, Canetti, Kianski & Zisk-Rony (2015) Israel	-	Parents were involved in phases of reintegration.	-	Psychiatrists, nurses, psychologists, social workers and nutritionists.	Anyone admitted to the psychiatric inpatient unit with an eating disorder diagnosis.	54% of participants had co-occurring depression. No further information was reported.	-
Eisler, Simic, Fonagy & Bryant-Waugh (2022) UK	-	Family therapy was a common treatment intervention offered, which assumes family involvement. A family-oriented philosophy to services was a key aim of the workforce training program.	-	Multidisciplinary team included both medical and non-medical staff with significant eating disorder experience. Given the national rollout of the service model across England, a comprehensive and coordinated workforce development and training package was offered to all teams within the service models. This included training in treatment models, supporting the development of a positive service culture, setting up supervision structures and fostering a culture of evidence-informed practice by promoting ongoing learning, keeping up to date with evolving evidence and routine monitoring of outcome and feedback data.	Enable direct access through self-referral or from primary care services (bypassing generic community mental health teams). Urgent referrals are to be responded to within 1 week, and routine referrals are to be responded to in 4 weeks.	The service model was required to provide interventions to treat the most common co-occurring mental health problems such as depression and anxiety. The types of treatment offered and how this was done were not reported.	During the roll out of these new service models, each team was grouped with two other teams located close by geographically to promote learning and sharing between the teams.
Fenner & Kleve (2014) UK	-	A family focussed treatment approach was used for most people (primary model family based treatment).	-	Clinical psychologists, specialist nurse therapist, psychiatrist and systemic family therapist. Also had access to an ‘extended team’ of paediatricians, general practitioners and dietitians Team members met every 6 weeks to review practice, for peer supervision and to keep up to date with emerging research.	Early referral encouraged. Referrals were assessed within 2 weeks.	-	-
Goldstein, Peters, Baillie, McVeagh, Minshall & Fitzjames (2011) Australia	-	Two parent only groups offered, and a weekly join psychology group. Parents were supported in setting and reviewing goals for their child, and taught skills to improve communication. Siblings were able to attend two sessions that aimed to help them understand the illness and its impact on the family.	-	Included nurses, dietitians, clinical psychology and occupational therapy.	People were primarily referred from outpatient service models (85.7%). To be able to access the service model, people need to be medically stable. BMI was not considered a factor when determining access.	Major Depressive Disorder was reported as a co-occurring condition for 17.9% of participants. Treatment of co-occurring conditions not reported.	-
Hayes, Tweedy & Chapman (2024) UK	Personalised treatment plans, considering the individual formulation and treatment progress.	New model had a much stronger family-centered approach. Parents and carers are offered skill-based meal coaching sessions and online or telephone support. An online four-week psychoeducation group is also available.	-	Multidisciplinary team led by nursing staff and supported by therapeutic care workers and a family therapist. Team also receives support from family therapists and psychologists in the outpatient service.	Not reported however the service model provides step-up care from the outpatient service and step-down care from the inpatient unit.	-	Service model sits within a wider eating disorder service system and the new model is integrated with the outpatient eating disorders service model.
Herpertz-Dahlmann, Borzikowsky, Altdorf, Heider, Dempfle & Dahmen (2021) Germany	Individualised treatment plans with the young person and their family.	Families offered psychoeducation focused groups and separate and conjoint family sessions. Young people and their families were visited at home and weekly family therapy was offered. The first two months of the service model focused on supporting parental management of food intake and other eating disorder symptoms.	-	Multidisciplinary team included a nurse, nutritional therapist, occupational therapist, psychotherapists, child and adolescent psychiatrist.	All young people were admitted to hospital prior to accessing the service model. A two-step admission process included an initial assessment at admission and a second assessment of final eligibility after 4-8 weeks of inpatient treatment. Inclusion criteria were a diagnosis of AN or AAN, aged between 12-18 years old, living with at least one carer and within a 60-minute commute of the treatment centre. Exclusion criteria were organic brain disease or severe psychiatric disorders, substance misuse, self-injurious behaviour, low intelligence, severe comorbid somatic disorders, insufficient knowledge of the German language, planned residential treatment, persistent severe eating disorder behaviour (including nasal gastric tube feeding or daily purging), serous somatic or psychiatric comorbidity or insufficient weight gain.	Many participants had a co-occurring psychiatric condition (n=18, 81.8%), however, it was not reported if these were treated at all. Severe co-occurring psychiatric conditions were an exclusion criteria for the service	Service model was closely linked to the hospital inpatient unit.
Johnson, Cook, Cadman, Anderson, Williamson & Wade (2022) Australia	-	-	-	Multidisciplinary team made up of general practitioners or primary care clinicians, private mental health clinician (psychologist or social worker) and optional dietitian.	2 weeks between identification and commencing treatment. A care coordinator role supported system navigation and ongoing coordination of the care team. Eligibility criteria included a DSM-5 eating disorder diagnosis.	-	Integration between primary care providers and treatment providers.
Kaplan, Hutchinson, Hooper, Gwee, Khaw, Valent & Willcox (2024) Australia	Mentions person-centred care.	-	-	Multidisciplinary team included dietitians, nurses and psychiatrist. All staff received 3 or 4 training modules in eating disorders, covering different aspects of eating disorder care and support.	All admissions screened using the SCOFF questionnaire. If a positive result, person would receive further assessment. If disordered eating behaviours or an eating disorder detected, the person was placed on pathway. Eligible with AN, BN or OSFED diagnosis.	All participants had a primary mental health condition, and co-occurring ED or disordered eating behaviour.	Integrated with existing general mental health inpatient units.
Milton, Hambledon, Dowling, Roberts, Davenport & Hickie (2021) Australia	Online service model that provided a personalised experiences to people seeking support.	Family and carers can independently contact the service model for family-oriented support.	Participatory design workshops embraced co-design principles, ensuring people with lived experience were able to contribute to the design and development of the online/telehealth service model.	Service model staffed by professionally trained and experienced counsellors.	Freely accessible to anyone. People can contact the service model via phone or online. Available 7 days per week during operating hours and a person should receive immediate support. Service offered immediate web based assessment (using a self-report questionnaire), with a dashboard of results provided.	-	Functions to connect people with external service models that can provide assessment and treatment for eating disorders. Can also be used as a platform where the care team can collaborate and share information between themselves (including the person with an eating disorder).
Moron-Nozaleda, Yanez, Camarneiro, Gutierrez-Priego, Munoz-Domenjo, Garcia-Lopez, Garcia, Garcia, Trujillo, Faya & Graell (2023) Spain	Individualised treatment plan devised with young person and their family. Aim to support person in least restrictive environment.	Had a family focus, with parents and carers being active treatment partners. Family were provided with psychoeducation, support (in person and via phone calls).	-	Consisted of psychiatrist, clinical psychologist, nursing team and paediatricians. Team met twice daily to coordinate cases and participated in weekly case discussion for more complex situations or presentations.	Most people were assessed within 48 hours of referral. Eligibility criteria included a child or adolescent with a severe eating disorder diagnosis (requiring hospitalisation), commitment from the young person to participate in treatment, 24/7 availability of at least one carer, a commute of 30 minutes or less from the hospital and parent or guardian agreement to participate in the treatment. Exclusion criteria were being medically unstable, extreme compensatory or purging behaviours, refusal to eat, severe suicide risk, severe risk of aggression and/or families with limited availability for home care.	-	Links with an eating disorder inpatient unit and outpatient treatment team/s mentioned.
Munro, Thomson, Corr, Randell, Davies, Gittoes, Honeyman & Freeman (2014) UK	Emphasis on open, transparent assessments and collaborative treatment planning at all stages.	-	-	Consultant psychiatrist, consultant clinical psychologist, clinical psychologists, clinical associate in applied psychology, dietitians, nurse, assistance psychologists and an administrator.	Weight based criteria for access (BMI 13 or less, OR BMI 15 or less and losing > 1kg per week), Person also had to be safe for community management.	-	The service model was one component of a service system in the region consisting of Tier one services that included guided self-help and internet based interventions, Tier 2 which was community based outpatient treatment, Tier 3 which was the current service and Tier 4 which was hospital inpatient unit.
Newell (2023) UK	Mentioned delivery of personalised care. Treatment is not limited by biological age. The all-age model enables continuity of care.	Family-oriented approaches and treatment models are part of the service model.	Consultation took place with people who had accessed the service (and their families) and undergone transition between child and youth and adult teams for ongoing treatment.	Multidisciplinary team initially consisted of a consultant psychiatrist, family/systemic therapist, nurses, dietitians, occupational therapist and psychological therapist. Team expanded as service model has evolved but disciplines within the expanded team were not reported.	-	-	Aimed to integrate adult and child and youth eating disorder community teams.
Newton, Bosanac, Mancusa & Castle (2013) Australia	A jointly developed and shared conceptualisation of the person's predicament was completed that included goals of treatment.	Aimed to engage both the person and their family to work collaboratively.	Feedback from the eating disorder inpatient unit consumer and carer advisory group and Eating Disorders Victoria (a lead non-government organisation providing support, information, and advocacy) supported the development of the service model. Consumer and carers were consulted as part of the development of the service model.	A total of 4.2 full time equivalent clinical staff were employed across medical, nursing, dietetic, psychology, social work and occupational therapy.	Designed to facilitate easy access and was available to all people over the age of 16 years old. There were geographical limitations as to who could access the service model. Any person who contacted the service was provided with advice on next steps. If a person had a medical referral and met inclusion criteria, an assessment was completed over four sessions.	Co-occurring conditions were identified, but it was not reported if these were treated within the service model. Most common co-occurring conditions included depressive episode (n=99, 50.3%), recurrent depressive disorder (n=26, 13.2%) and dysthymia (n=34, 17.3%).	Aimed to develop, maintain and maximise partnerships with private and public care providers to deliver a continuum of care for eating disorders.
Painter, Ward, Gibbon & Emmerson (2010) Australia	-	A 6-week carer program was delivered in partnership with another organisation.	-	One full time manager and 3.5 full time equivalent specialist clinicians (nursing, dietetics, social work and psychology).	Key area of the service model was streamlining intake to existing eating disorder service models (such as the eating disorder inpatient unit). Intake service also provided resources and ongoing support to referrers and advocacy for people accessing public health services for an eating disorder.	-	Enabled integration and improved processes between the system of care for eating disorders, particularly the metropolitan based inpatient and community eating disorder services.
Penfold (2015) UK	-	-	-	Nurse led service model, with psychological and psychiatry input. External dietitian provides regular groups and sessions.	Timeframe between referral and commencing treatment is not reported. Day program was accessible to adults aged 16 years or older, with a diagnosis of anorexia nervosa.	-	Day program sits within the wider eating disorder service system, and was established to reduce hospital admissions and provide more intensive community support (including outreach). Established referral paths from primary care, with most referrals being received from general practitioners. Mental health services can also refer.
Simic, Stewart, Eisler, Baudinet, Hunt, O’Brien & McDermott (2018) UK	-	Families were included in treatment to mobilise family resources	-	Multidisciplinary team included psychiatrists, a paediatrician, psychologists, nurses, family therapists, an art therapist and a dietitian.	Adolescents (aged 11-18 years old) could access the service model if they had experienced rapid weight loss for longer than 4 weeks, or remained static below 80% of their expected BMI for more than 4 weeks. The service model also helped people step down from inpatient units.	Treatment of co-occurring conditions was not reported, but the results reported improvements in ratings of mood, and ability to regulate emotions.	Embedded into a comprehensive eating disorder service system.
Strand, Gustafsson, Bulik & Hausswolff-Juhlin (2015) Sweden	Self-admission is based on the premise that a person can make a choice as to when they are admitted, without needing to explain or justify their need.	-	-	-	People eligible for the service model could access admission immediately, if a bed was available. To be eligible, people must have been admitted in the past 3 years and be receiving ongoing treatment for an eating disorder with the wider service system. Exclusion criteria were active suicidal or self-injurious behaviour, active substance use. No BMI criteria was applied.	-	Service model sat within the existing inpatient unit, and within the wider eating disorder service system.
Suetani, Yui & Batterham (2015) Australia.	-	Clinical team held meeting with family within 48 hours of their child’s admission.	-	Multidisciplinary team noted, but not further described.	Children (<18 years old) admitted to the unit with an eating disorder diagnosis.	-	Eating disorder service model overlaid onto the general paediatric hospital ward (as a result of increasing eating disorder admissions over a number of years). Links with child and adolescent mental health services for psychological support while on ward and in preparation for ongoing treatment after discharge.
Tantillo, Starr & Kreipe (2020) USA	-	-	Included people with lived experience (parent peer mentor and young person peer mentor) who contributed to the delivery of education, consultation and support.	Eating disorder experts (n=11) formed the team. Professionals included nursing, adolescent medicine, psychiatry, art therapy, dietetics, psychology, care management, parent peer mentor and young adult peer mentor.	Clinicians wanting to access the service model had regular weekly opportunities to meet via telehealth.	-	Aimed to support already established health services, particularly those who do not have expertise in eating disorders.
Tchanturia, Smith, Glennon & Burhouse (2020) UK	-	This was recognised as an area where ongoing work is needed.	Many aspects of the service model were co-designed with people with lived experience. Pathway were developed using co-design principles and ongoing input.	Multidisciplinary team not explicitly discussed, several training and professional development activities (with a focus on AN and autism) for the wider eating disorder service system workforce mentioned.	All people with AN who access the wider service system were screened for autism, and a positive result would trigger a more in-depth assessment to ascertain diagnosis and eligibility. Service model not available to other eating disorder diagnoses or child and youth.	The service model offered treatment for AN and Autism as cooccurring conditions.	Service model sits within the wider eating disorder specialist service system.
Wallis, Alford, Hanson, Titterton, Madden & Kohn (2013) Australia	A comprehensive family assessment informed the development of treatment that was specifically tailored to each family.	Families were essential and the whole family was admitted and part of the treatment.	Service model was developed following suggestions received from families.	Multidisciplinary team consisted of a child and adolescent psychiatrist, paediatrician, clinical nurse consultant, nursing staff and family therapists. The team had support from dietetics, physiotherapy and the hospital school.	Targeted families such as those from regional and remote areas, families with children under the age of 12, families with limited support and families with complex relational or illness dynamics. Admission criteria were: child or adolescent were under 18 years old, primary diagnosis of an eating disorder, young person had been medically stable for at least 72 hours prior to admission and the young person is eating.	-	The service model was part of a wider service system offering hospital and outpatient eating disorders treatment.
Weber & Davis (2012) Australia	-	Consultations were offered to family and carers.	-	One part time social worker.	People could self-refer, plus referrals came from primary care providers, local universities and schools. People were eligible if they were aged over 14 years old.	-	Assessment and referral service model aimed to connect people (following diagnosis of an eating disorder) to treatment providers.
Williams, Dobney & Geller (2010) Canada	Is person-centred and allows people to set their own goals (rather than the clinical team). There is capacity for outreach and focus on quality of life rather than recovery. Pace of treatment is determined by the person.	-	A first step to developing the service model was focus groups were held with people with longstanding eating disorders to explore what they thought would be beneficial.	Multidisciplinary team includes staff from a hospital-based eating disorder team and a community-based mental health rehabilitation team. Specific disciplines of the team included outreach counsellors, case managers, family therapists, medical internist, nurse, psychiatrist, dietitian.	-	-	-
Williams, O’Reilly & Coelho (2020) Canada	-	Family involvement offered, and family and carers were viewed as integral to the support of people in the program. Ultimately, people attending the program could decide if they wanted the involvement of family or carers.	-	Team includes medical, psychiatric, nursing and allied health professionals	Referrals are made by community specialised eating disorder services or mental health teams, or from a primary care provider. Eligibility criteria included aged between 16-24, and being medically and psychiatrically stable.	-	Service model is part of an integrated provincial network (service system) for people with eating disorders.

*AN* Anorexia nervosa, *AAN* Atypical anorexia nervosa, *ARFID* Avoidant restrictive food intake disorder, *BN* Bulimia nervosa, *BMI* Body mass index, *DBT* Dialectical behaviour therapy, *DSM V* Diagnostic statistical manual of mental disorders fifth edition, *FREED* First Episode and Rapid Early Intervention for Eating Disorder Service, *OSFED* Other specified feeding and eating disorder

### Data analysis

Descriptive statistics, a narrative summary and tables were used to present the findings in relation to the questions the scoping review aims to answer.

## Results

Table 2 presents the 30 literature sources included in this scoping review. There were 11 quantitative studies, three qualitative studies and five mixed methods studies. Retrospective chart reviews or audits were commonly used in both quantitative and mixed methods studies [26–32]. Several studies explored the feasibility and acceptability of the service model [7, 30, 33, 34]. While some studies sought to understand the outcomes achieved by people accessing the service model [28, 29, 31, 35, 36], only one reported if the outcomes were sustained at 6 months or more [37]. Overall, there was a notable absence of high-quality quantitative studies such as comparative studies and randomised control studies. Qualitative studies focused on understanding the experience of service models [38, 39] or sought participant input into the design of a technological web-based service model [40]. A further 11 studies did not include an evaluative or research component and solely described a service model.

The literature sources originated from predominantly Western countries, including the UK (n = 11), Australia (n = 9), USA (n = 3), Sweden (n = 2), Canada (n = 2), Spain (n = 1), Israel (n = 1) and Germany (n = 1).

Most service models described were eating disorder specialist (n = 27). Only three [26, 27, 31] literature sources documented a service model that was not eating disorder specialist but provided treatment to people with an eating disorder. Table 2 provides details on the types of service models included in this review.

Table 3 summarises the key characteristics of service models included in this review, under the headings of person-centred care, involvement of family and carers, co-design and lived experience contribution, multidisciplinary team, accessibility, identification and management of co-occurring conditions and integration with the service system. A narrative summary of the different types of service models followed by key characteristics of eating disorder service models is discussed below.

### Eating disorder service models: a narrative summary

Most service models included in this review were eating disorder specialist (n = 27), meaning that they solely focused on supporting the health needs of people with eating disorder diagnoses. Service models were primarily located in metropolitan areas (n = 17) [7, 26–30, 32, 35, 37, 41–48] with some located in regional areas (n = 7) [31, 34, 36, 38, 49–51] and one in a rural location [39]. An additional five service models provided health services across metropolitan, regional and rural locations [52–54], including telehealth service models [33, 40] (see Table 2).

Children and adolescents were the focus of 11 service models, [26, 29–31, 34, 35, 37, 42, 45, 47, 53], while a further three service models targeted young adults (see Table 2). One service model included people from the age of 4 to 21 years [28]. Two service models accepted people over the age of 14 years [36, 39] and another two were accessible to people over the age of 16 years [43, 49]. Only four service models reported an adult focus [38, 44, 46, 52]. Another four service models were available to people of any age, [33, 40, 41, 50] and three did not report specific details on the age range of the target population [27, 48, 51] (see Table 2).

Some service models provided treatment only to people with specific diagnoses. For example, people with anorexia nervosa (n = 4) [29, 47, 49, 51], a ‘restrictive’ eating disorder (n = 1) [37], anorexia nervosa and atypical anorexia nervosa (n = 1) [34], avoidant restrictive food intake disorder (ARFID) (n = 2) [28, 42] or anorexia nervosa and autism (n = 1) [46]. Others (n = 8) explicitly stated that they accepted people with any eating disorder diagnosis into the service model [7, 26, 36, 38, 41, 45, 53, 54]. People experiencing longstanding eating disorders, or who were unresponsive to usual treatment were the focus of two service models. One of these enabled self-admission to hospital [44] and the other focused on improving a person’s quality of life [48] (see Table 2).

Predominantly, service models were based in community settings (n = 17). There were nine service models that provided community based outpatient care [31, 36, 38, 39, 41, 42, 48, 50, 53]. Another group of community based service models offered intensive support (n = 7) such as day programs, outreach or hospital in the home [29, 30, 34, 35, 37, 49, 51]. Early intervention was the focus of one community service model that was described in two separate studies [7, 54]. This service model targeted young adults within three years of their eating disorder diagnosis. Service models within hospital service settings (n = 7) encompassed one that targeted people with a diagnosis of anorexia nervosa and autism [46], one that offered a self-admission program [44], three that aimed to provide staged, consistent hospital care, [26, 28, 45] and a family admission program [47]. An additional hospital based service model sought to implement a pathway that facilitated eating disorder treatment and support for people with a primary mental health diagnosis in a psychiatric hospital with a co-occurring eating disorder [27].

There was one service model in a residential setting [32] and a further two were telehealth service models [33, 40].

Finally, two service models covered multiple service settings. One [52] included community, community intensive support and hospital service settings over a large geographical region. A service model described by Newton et al. [43] offered outpatient community based treatment and an intensive community based day program.

### Service model characteristics

#### Person-centred care

Person-centred care was a characteristic of service models identified in 16 sources (see Table 3).

The most common reference to person-centred care was in relation to aspects of treatment (such as goals or the treatment plan). These were reported to be collaboratively developed between the clinical teams, the person with an eating disorder and sometimes, their family and carers [7, 27, 29, 30, 34, 42, 43, 51]. Only three service models [38, 44, 48] reported person-centred care that extended beyond this collaboration. One service model provided support to people who did not respond to usual treatment for eating disorders. The focus of this service model was quality of life. People using the service model could set their own treatment goals and choose their treatment intensity (how often they saw the team), highlighting a person-centred approach [48]. A second service model aimed to maximise choice and control for people by offering a person-centred ‘drop in’ self-referral [38]. This meant that people who felt they needed support for an eating disorder could attend the service and be seen immediately, instead of prearranging appointments. The third example was a service model that allowed people with an eating disorder (who were receiving treatment within the wider eating disorder service system) to request a brief self-admission to an inpatient unit when they felt they were at risk of relapse or needed additional support to manage their condition [44].

#### Involvement of family and carers

Family and carer involvement was identified in many service models (n = 22) (see Table 3). Family and carers were often part of the treatment approach, for example in family-based treatment or multi-family therapy [30, 31, 41, 47, 50, 53]. Additional day patient program service models (n = 2) also involved families in treatment, aiming to help families feel more confident to support their child's treatment and recovery [35, 37]. Other intensive outreach and hospital in the home service models (n = 3) also required the involvement of family or carers to facilitate treatment delivery and/or monitor a person’s safety in the community [26, 29, 30, 34]. A service model for young people hospitalised with ARFID provided families with education and guidance about meal support and involved families in discharge planning [28].

Family involvement was ‘encouraged’ by four service models (including one with two sources) [7, 32, 42, 43, 54] but limited additional details were provided. In one case, family involvement was dependent on whether the person with the eating disorder agreed to family involvement [32]. Other ways family and carers were included in service models were through clinical meetings [45], or through the provision of a structured carer-focused intervention [52]. The service model described by Tchanturia et al. [46] recognised a need to improve family involvement and noted that this was a focus of ongoing work.

#### Co-design and lived experience contribution

Co-design principles were reported to be used in the development of two service models [40, 46]. In one instance, people with lived experience helped design a service model for people with anorexia nervosa and autism, with a commitment to seeking ongoing input from them [46]. In another instance, participatory design workshops provided an opportunity for people with lived experience to contribute to the service model design and development [40].

After the ‘First episode and Rapid Early intervention in Eating Disorders’ (FREED) service model was developed, a group of young people who had benefited from or supported FREED was formed. They contributed to new initiatives for promoting and expanding the service model [7, 54].

Other service models were conceptualised using feedback from families [47], consumer advisory groups [43] or used consultation from people who had previously used eating disorder services [50] to inform service model development. For a service model treating people with longstanding eating disorders, insights from focus groups with people with lived experience helped shape its design [48].

One service model reported that people with lived experience were part of the team who delivered healthcare services [33].

#### Multidisciplinary team

Most service models (n = 25) included a multidisciplinary team (see Table 3). However, aside from reporting on the disciplines included within the team, other detailed information about the team (such as if staff were full-time or part-time and their level of seniority) was not provided.

Some service models stated they had a multidisciplinary team [45, 46], while others specified their team included medical and non-medical clinicians with eating disorder expertise [53] or consisted of a primary therapist and physician [41]. Other service models provided more detailed information about the disciplines within the team (n = 20).

The most common disciplines included nursing (n = 19) [26–35, 37, 38, 43, 47–52], psychology and psychotherapy (n = 16) [26, 28–31, 33–38, 43, 49–52], psychiatry (n = 14) [26–28, 30, 32–34, 37, 38, 47–51] and dietetics or nutrition (n = 13) [26–28, 33–37, 43, 48, 50–52].

Less frequently reported disciplines included medical professionals (including physician, adolescent medicine specialist and gastroenterologist) (n = 6) [28, 32, 33, 38, 43, 48], social workers (n = 6) [26, 28, 36, 38, 43, 52], family therapists (n = 6) [29, 31, 37, 47, 48, 50], occupational therapists (n = 5) [34, 35, 42, 43, 50], paediatricians (n = 3) [30, 37, 47], art therapists (n = 2) [33, 37] and speech therapists [42].

In addition, an online telehealth service model was staffed entirely by trained counsellors [40]. Another included team members with lived experience of an eating disorder [33]. A third, described by Johnson et al. [36] involved general practitioners and primary care practitioners.

Training and development for team members were described in some service models. For example, a service model for people with anorexia nervosa and autism included workforce training and group huddles to boost staff knowledge and confidence [46]. Similarly, a hospital based service model introduced training modules for all staff, helping to implement the new service model and improve care for those with a primary mental health diagnosis and co-occurring eating disorder [27]. Eisler et al. [53] also described a training curriculum that facilitated coordinated upskilling of a large national workforce in England (approximately 900 people). The training program provided education on eating disorders, evidence-based treatment models, supervision, provided train-the-trainer opportunities and regular updates on new research [53]. Other service models (n = 3), recognised the value of providing high-quality supervision [41, 50] and holding regular meetings to review clinical practice and emerging research [31].

#### Accessibility

Most of the service models (n = 27) provided information about access to the service model, with some of these (n = 17) outlining inclusion and/or exclusion criteria (see Table 3).

Facilitating access to eating disorder service models included responding to referrals and conducting an intake call within 24 h [41] or 48 h [7, 30, 54]. Reducing the time between referral and assessment was a focus for four service models, with one model being described in two separate sources [7, 31, 36, 53, 54]. The child and adolescent service model described by Eisler et al. [53] responded to urgent referrals within 1 week, and ‘less urgent’ referrals were responded to within 4 weeks. Other service models [7, 31, 36, 54] reported a 2-week timeframe between referral and assessment for people eligible to access the service model.

Another service model overcame delays between referral, assessment and/or commencement of treatment by not having a waiting list [41]. Self-referral was possible for five service models [38, 39, 42–44], however, one required a medical referral before an assessment could be completed [43]. An early intervention service model described by Brown et al. [7] and later Allen et al. [54], initially did not accept self-referrals but identified that later versions of the model planned to.

The criteria that determined access to each service model varied in detail. For example, an early intervention service in the UK required people to have received a diagnosis of an eating disorder within the last three years [7, 54]. Weight-based criteria such as rapid weight loss or weight below a particular threshold were reported for two service models [37, 51]. Another two service models specified that there were no weight criteria for access [35, 44], but had other criteria determining access. For example, the service model described by Strand et al. [44], required people to be engaged in eating disorders treatment and to have had an inpatient admission in the previous 3 years. An additional three service models reported no criteria for access at all [38–40], meaning anyone who thought they had an eating disorder could access the service model for assessment. A primary eating disorder diagnosis (any eating disorder) was explicitly stated as an inclusion criterion for many service models (n = 12, including one with two sources). A specific type of diagnosis, for example ARFID, was a requirement for other service models [28, 42]. One service model targeted specific co-occurring conditions (anorexia nervosa and autism) [46]. One service model required an eating disorder diagnosis but excluded individuals with extreme eating disorder compensatory behaviours (such as purging or refusing to eat) [30] and another was unable to accept people with a ‘severe’ eating disorder [34]. There were two service models requiring people accessing the service model to be able to eat food (i.e. not being fed via a nasogastric tube) [30, 47].

Being medically unstable was an exclusion criterion for five service models [7, 30, 32, 35, 47]. Other service models stipulated that a person needed to ‘be safe’ (without defining what safe meant) for treatment in the community [7, 51] or required a person to be psychiatrically stable [32]. People with a planned admission to a residential unit were unable to access one service model [34].

Other exclusion criteria reported by service models included suicidal ideation [30, 44], people with a high risk of aggression [30], substance use or self-injurious behaviour [44]. Co-occurring conditions, including learning disabilities, low intelligence or organic brain disorders, prevented access to two service models [7, 34].

An unwillingness to commit to treatment or a lack of readiness to change were other exclusion criteria [30, 38, 47]. For people to access to some service models, a parent or carer needed to commit and be available to support treatment [30, 34, 47]. Furthermore, living outside a designated geographical area prevented access to three service models [30, 34, 43].

#### Co-occurring conditions

Co-occurring conditions were reported for participants of six service models [26, 28, 34, 35, 42, 43]. However, it was unclear whether the service model provided treatment for co-occurring conditions. Two service models offered treatment for (some) co-occurring mental health conditions alongside an eating disorder, but did not include further detailed information [41, 53]. In contrast, in a hospital service model for people with general mental health conditions, eating disorders were treated as the co-occurring condition [27].

Another service model recognised a different approach was required for a specific set of co-occurring conditions (anorexia nervosa and autism) [46].

#### Integration with the service system

Fifteen (including one with two sources) service models were integrated within a service system. Most (n = 12, including one with two sources) eating disorder specialist service models were part of a wider eating disorders service system (see Table 3). One service model sat within a paediatric inpatient service setting and reported connections with child and adolescent mental health service models [45]. Other service models (n = 2) reported established partnerships with external organisations or parts of the health system (such as between private and public service models, or with academic institutions) [41, 43].

Some service models (n = 3) aimed to improve cohesion and connection between other service models and/or settings within the service system. For example, enhancing pathways between primary care providers (who often identify or diagnose eating disorders) with service models that could provide appropriate, evidence-based treatment [36, 39, 52]. One service model aimed to integrate treatment and support in an inpatient setting for people with co-occurring mental health conditions and eating disorders to optimise outcomes [27]. Providing navigation and help-seeking support to people seeking help for an eating disorder was a key function of a national telehealth service model [40]. Another service model facilitated knowledge sharing between experienced and less experienced clinicians, to improve treatment for people with eating disorders [33].

Integrating two existing service models created a new one, allowing children, youth and adults to receive treatment in one place, improving continuity of care [50].

## Discussion

This scoping review aimed to describe the types of service models for eating disorders and their characteristics. A range of service models for eating disorders were included showing significant differences in design, setting, purpose and target population.

Most service models for eating disorders were based in the community and were specialist eating disorder services, providing assessment and treatment exclusively to people with eating disorders. While several advantages of specialist eating disorder service models have been identified in the literature, their capacity to offer assessment and treatment for co-occurring conditions, often seen alongside eating disorders, remains unexplored. This is concerning when a recent rapid review found people with eating disorders have high rates of medical and psychiatric co-occurring conditions [55].

There were 17 service models within this review that outlined eligibility and/or exclusion criteria people had to meet to gain access. Extensive eligibility criteria can hinder access to service models [56] and exclude people with complex eating disorder presentations [57]. Given the stringent eligibility criteria of most specialist eating disorders service models, it is likely that many individuals with eating disorders are prevented from accessing the care they need.

Simplified access to service models is key to ensuring early intervention of eating disorders. Early intervention is important because it improves the likelihood of recovery from an eating disorder [9, 58]. Early intervention is crucial for reducing the ‘duration of untreated illness’, which is the time between the onset of an eating disorder and the start of treatment [9]. Research suggests the average duration of untreated illness is 5.28 years [59]. Barriers such as ‘inaccessible treatment’ [59] or strict and rigid eligibility criteria may contribute to this delay [56, 57]. Because delayed treatment leads to poorer health outcomes for people with eating disorders [4], improving access to service models is essential.

Potential solutions to improve service model access were identified in this review. Solutions included promoting informal, self-referral entry points [38, 39, 42–44] and prioritising intake and assessment when a person has a suspected eating disorder [7, 42, 43, 54]. Removing waiting lists was another strategy identified [41], but unless a person can easily access help from another service model this may still result in difficulty with access and treatment delay. Some evidence suggests that people put on a waiting list may be less likely to commence treatment or more likely to drop out of treatment [60, 61].

Recent evidence suggests that effective integration of service models also promotes access to healthcare [62], improves a person’s outcomes, and enhances the efficiency of the service system [63]. Within this review, some service models had existing pathways between other eating disorder service models, supporting continuity of care and forming a service system. Coordinated integration of multiple service models across different service settings can create a stepped care service system [5]. This is promising, as advantages of stepped care service systems have been described in the literature [4] and are a recommended framework for eating disorders service systems in Australia, Ireland and Canada [5, 15, 16].

Only a few service models had established care pathways and integrated with service models outside the eating disorder sector, for example with paediatric service models [45] or with primary care providers [36]. Cross-sector service model integration is complex and difficult to do [63] with no clear or transferable guidance [64]. However, cross-sector integration can offer a solution to effectively addressing eating disorders and co-occurring conditions. While limited examples of cross-sector service model integration were found in this review, they are promising as they demonstrate that overcoming challenges with level of integration is possible [12].

This review highlights the benefits of community based service models, which aligns with recent evidence showing that contemporary mental health and eating disorder service models are primarily community based [65]. There are multiple benefits to eating disorder service models in the community. For example, a person can remain at home, remain connected to family and friends and participate in their usual routines [4, 65]. Recommended psychological therapy approaches for eating disorders (such as Cognitive Behaviour Therapy-Enhanced and Family Based Treatment) are mainly designed for delivery in community settings [66, 67]. Health care provided in the community is also more cost-effective and promotes recovery [4, 65].

Over half of the service models described in this scoping review mentioned person-centred care as an essential characteristic of the model. Person-centred care is treating people as individuals with unique needs and circumstances [12]. While person-centred care is more akin to an approach to service delivery than a characteristic of a service model, this was a key finding of this scoping review. Literature on person-centred care suggests that incorporating the approach into service models can be challenging yet crucial to enhance a person’s experience of service models and their health outcomes [12, 68]. In this review, it was difficult to ascertain how person-centred care was embedded into many service models. However, three service models included a detailed explanation of how person-centred care was prioritised [38, 44, 48]. One of these was the community outreach eating disorder specialist service model described by Williams, Dobney and Geller [48]. The focus on quality of life and autonomy given to people accessing this service model aligns with person-centred care for serious illness, identified as a construct in a systematic review by Giusti et al. [69]. In that systematic review, person-centred care included person and family empowerment and autonomy, and treatment that has a quality of life focus [69]. Further emphasising the value of person-centred service models, a recent meta-synthesis of 22 studies from eight countries found participants with a lived experience of an eating disorder viewed person-centred care as crucial in treatment [70]. Research conducted on a person-centred eating disorder service model included in this review [48] found improvements in participants body mass index, eating disorder symptoms and feelings of hopelessness. These results suggest that person-centred service models can lead to improved outcomes for eating disorders.

Interestingly, family-empowerment and autonomy, identified by Giusti et al. [69] as part of person-centred care, was mentioned in 22 service models included in this review. This suggests that involving family and carers is a key characteristic of service models in eating disorders. The use of family-centred approaches, such as family-based treatment, was a common way families and carers were included in models described in this review. This aligns with findings from a recent Australian report evaluating the impact of the Medicare Benefits Schedule items. This report found that families and carers should be involved in treatment for a loved one with an eating disorder [71]. Other ways family and carers were incorporated in service models was through the provision of education and coaching, and collaboration on treatment goals and discharge planning. Involving family and carers in decisions about treatment and recovery from an eating disorder is crucial [72]. This is because family and carers are often essential drivers of treatment [73], and frequently have a role in supporting a person emotionally and financially [74, 75]. A recent scoping review confirmed that providing education to families and carers of adults with eating disorders may improve their loved one’s outcomes [76]. Involving families and carers in service models is often necessary, however, there is scope to better understand and recognise carers’ needs, experiences and perspectives beyond their involvement in treatment and provision of education.

Co-design is one way that people with a lived experience of eating disorders (and their families and carers) can be involved in the design, development and/or delivery of a service model [77]. The advantages of co-design include more effective resource allocation, innovative solutions to complex problems and the development of new knowledge [77]. Given these advantages, it is surprising that only two of the service models identified in this review incorporated principles of co-design [40, 46]. This may be explained by challenges associated with co-design in the field of eating disorders, such as stigma and potential increased risk of relapse by being exposed to eating disorder related content [78]. More broadly in the field of eating disorders, co-design appears to be gaining traction with the emergence of co-designed treatment approaches [79] and co-designed educational programs for the eating disorder workforce [80]. Given the lack of co-designed service models, it is a timely opportunity for the sector to invest and promote the involvement of people with lived experience in this area.

The recent introduction of a lived experience workforce to the multidisciplinary team in eating disorder service models is a significant advancement in the field. This workforce was part of only one telehealth service model included in this review [33], despite the documented recognition of the valuable contribution the lived experience workforce can make [81, 82]. Furthermore, the composition of the multidisciplinary team, and disciplines within it, varied significantly across eating disorder service models. Multidisciplinary teams are well established as best practice in eating disorders [4, 66, 83]. However, recent research in eating disorders has indicated that the value of multidisciplinary teams is enhanced with interprofessional collaboration. According to WHO, interprofessional collaborative practice occurs when members from a multidisciplinary team work together with people, their families and others to deliver high quality care [84]. A qualitative study conducted in Australia found that interprofessional collaborative practice provided to people with eating disorders improved their treatment satisfaction, engagement and outcomes [85]. Multidisciplinary teams of service models in this review predominantly included nurses, psychologists and psychiatrists, suggesting there is an opportunity to increase the diversity and expertise within teams, to offer holistic, comprehensive care that addresses the breadth of a person’s recovery needs.

## Summary

In summary, service models for eating disorders are diverse. However, some important characteristics have been identified and discussed in this review. These characteristics are evident at a person level (person-centred care, involvement of family and carers), service model level (accessibility, the multidisciplinary team, co-design and lived experience contribution and identification and management of co-occurring conditions) and service system level (integration of service models). In the future, service models should be configured and designed with these fundamental characteristics in mind as the sector moves towards service models and a coherent integrated service system that is responsive and effective at providing the right support, at the right time, for people with eating disorders.

## Limitations, future directions and clinical implications

Thorough description, development and evaluation of service models are complex [86], which could explain why it is not frequently completed and reported in publications. A key focus for future research should be thorough evaluation of existing service models to understand their characteristics and how service models promote access to high quality assessment, treatment and recovery outcomes.

Service models in this review predominantly targeted children and young people, while significantly less focused on people with longstanding eating disorders, or people with co-occurring conditions. This signals a need to develop and evaluate service models that can respond to the diverse clinical populations requiring assessment and treatment for an eating disorder.

Completing a scoping review ensured that the breadth and extent of the literature on eating disorder service models and their characteristics were captured and not restricted to primary research studies [21]. However, some service models may not have been captured in this review. While every effort was made to develop a comprehensive search strategy, there may be search terms or terminology omitted. For example, some terms may differ between different countries, regions and healthcare systems. Furthermore, some service models may have been missed because their design and characteristics have not been documented or made publicly available. There were no studies included in this review from Africa, Asia or South America, which is a limitation of the review.

Of the studies included in this review, many collected data retrospectively from existing medical records. This indicates that future research is needed to capture outcomes before, during and after a person accesses an eating disorder service model.

There are several clinical implications relating to the findings of this review. Firstly, the importance and value of clinicians adopting and advocating for person-centred care is paramount. This includes treatment that addresses not only the eating disorder, but also any co-occurring conditions. Interprofessional collaboration can also improve person-centred care by leveraging the diverse perspectives and expertise of a multidisciplinary team. As clinicians, it is crucial to advocate for the wider adoption of co-designed service models and to include people with lived experience as key members of the eating disorder workforce.

## Conclusion

The results of this scoping review underscore the vast differences between eating disorder service models. These differences indicate the absence of agreed shared characteristics for service models in the sector. While acknowledging these differences, it is important to note that some key characteristics of eating disorder service models were identified. These were person-centred care, involvement of family and carers, co-design and lived experience contribution, a multidisciplinary team, accessibility, identification and management of co-occurring conditions and integration with the service system. In addition, and interestingly, this review uncovered innovative and, where a research component was included, effective service models that support recovery from eating disorders.

In this context of diversity, differences, and variations, this scoping review has contributed to the limited research available on eating disorder service models and their key characteristics.

## Data Availability

No datasets were generated or analysed during the current study.

## Abbreviations

ARFID: Avoidant Restrictive Feeding and Intake Disorder
FREED: First Episode and Rapid Early Intervention for Eating Disorders
NEDC: National Eating Disorders Collaboration
UK: United Kingdom
USA: United States of America
